# Change to movement and morphology of the median nerve resulting from steroid injection in patients with mild carpal tunnel syndrome

**DOI:** 10.1038/s41598-020-72757-2

**Published:** 2020-09-24

**Authors:** Hyunseok Moon, Byung Joo Lee, Donghwi Park

**Affiliations:** 1 Department of Rehabilitation Medicine, Daegu Fatima Hospital, Daegu, Republic of Korea; 2 Department of Physical Medicine and Rehabilitation, Ulsan University Hospital, University of Ulsan College of Medicine, 877, Bangeojinsunghwndo-ro, Dong-gu, Ulsan, 44033 Republic of Korea

**Keywords:** Musculoskeletal system, Neuropathic pain

## Abstract

There are conflicting hypotheses regarding the initial pathogenesis of carpal tunnel syndrome (CTS). One hypothesis characterizes it as inflammation of the median nerve caused by compression, while another hypothesis characterizes CTS as non-inflammatory fibrosis of the subsynovial connective tissue (SSCT). This study aimed to investigate the differences in the ultrasonography parameters before and after a steroid injection, which is effective for CTS, to elucidate the initial pathogenesis of CTS and the mechanisms of action of the injected steroid. Fourteen hands from 14 healthy participants and 24 hands from 24 participants with mild CTS were examined. Dynamic movement and morphology of the median nerve before and after steroid injection were measured. There was no significant difference in the normalized maximal distance of the median nerve, which reflects the degree of fibrosis in the SSCT indirectly, during finger and wrist movements before and after the injection among patients with CTS (p > 0.05). Among the parameters that indirectly reflects the degree of median nerve compression, such as normalized maximal change in the aspect ratio of the minimum-enclosing rectangle (MER), maximal change in the median nerve perimeter, and maximal value of the median nerve cross-sectional area (CSA), statistically significant differences were not observed between values of the normalized maximal change in the aspect ratio of the MER and maximal change in the median nerve perimeter, during finger and wrist movements recorded before and after the injection in patients with CTS (p > 0.05). However, multivariate logistic regression analysis revealed that the change in the normalized maximal value of the median nerve CSA, according to finger and wrist movement was correlated with the administration of the steroid injection (p < 0.05). In conclusion, compared to that noted before steroid injection, the median nerve CSA noted during finger and wrist movements changed significantly after injection in patients with mild CTS. Given the improvement in median nerve swelling after steroid injection, but no improvement in the movement of the median nerve during finger and wrist movements, median nerve swelling due to compression (rather than fibrosis of the SSCT may be the initial pathogenesis of early-stage (mild) CTS, and the fibrous changes around the median nerves (SSCT) may be indicative of secondary pathology after median nerve compression. Further studies are required to validate the findings of our study and confirm the pathogenesis of CTS.

## Introduction

Carpal tunnel syndrome (CTS) is the most common type of entrapment neuropathy of the peripheral nerves^1^. The diagnosis is usually based on the presence of characteristic signs and symptoms as well as findings from electrophysiological studies^2,3^.

There are conflicting hypotheses regarding the initial pathogenesis of CTS^4–8^. One hypothesis has characterized the syndrome as inflammation of the median nerve caused by compression, while another has characterized it as non-inflammatory fibrosis of the subsynovial connective tissue (SSCT)^12–15^. Several previous studies have suggested that reduced mobility of the median nerve in patients with CTS shares similar etiology^5,6^. Moreover, these studies have proposed that nerve compression may be secondary to the initial stiffness of the SSCT. However, other studies have suggested that inflammation of the median nerve secondary to its compression may lead to the development of CTS^7,8^. In a recent ultrasonography-based study, Park et al.^4^ have suggested that only nerve swelling-related parameters changed in the early stages of CTS (Bland’s scale 1–2) without significantly affecting the mobility of the median nerve during finger or wrist movements. The authors concluded that the deformation of the median nerve may be associated with the initial pathogenesis of CTS rather than SSCT fibrosis.

There are various treatment options for CTS, including administration of non-steroidal anti-inflammatory drugs (NSAIDs), wrist splinting, steroid injections, and surgery^9^. Among them, steroid injection is an easy, safe, and effective treatment that is frequently used^9^. The present study investigated ultrasonography parameters before and after steroid injection to elucidate the initial mechanisms of CTS pathogenesis and the mechanisms of action of the injected steroid. To this end, we investigated changes to deformation and displacement of the median nerve after intra-carpal steroid injection in patients with CTS.

## Methods

### Participants

The Institutional Review Board at the Daegu Fatima Hospital reviewed and approved the protocol for this investigation, and all participants or their legal guardians provided informed consent or assent prior to participation in the study (DFH16ORIO310). All study interventions and measurements were performed in line with the relevant guidelines and regulations.

**Figure 1:**
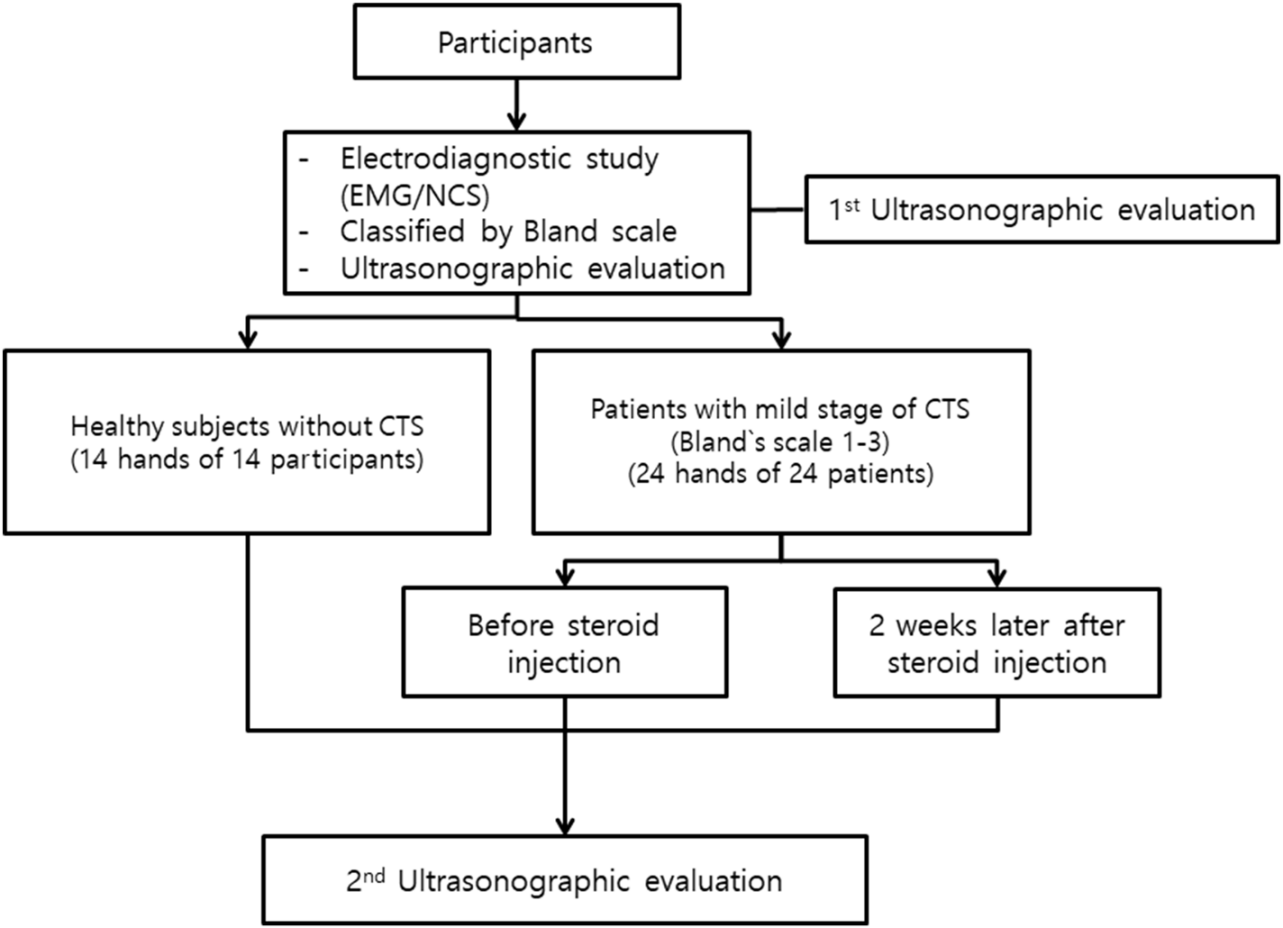
Flowchart of the study.

We analyzed 14 hands from 14 healthy participants (8 men) without any symptoms or evidence of peripheral neuropathy in an electrophysiology study. We also analyzed 24 hands from 24 patients with mild CTS^10^ (Bland’s scale 1–3^11^; 7 men), diagnosed and confirmed on the basis of an electrodiagnostic study. Patients with CTS were excluded if they met any of the following criteria: a history of injection around the median nerve; rheumatoid arthritis or other degenerative joint diseases in the hand or wrist; space-occupying lesions in the wrist; coexistent neurologic disease, such as polyneuropathy, proximal median neuropathy, cervical radiculopathy; thyroid disease or diabetes mellitus; a history of fractures or other trauma to the hand or wrist; and another systematic disease (Fig. 1).

### Nerve conduction study

A nerve conduction study (NCS) was performed using a Medelec Synergy device (CareFusion Corporation, San Diego, CA). A sensory and motor NCS of the median and ulnar nerve and sensory NCS of the superficial radial nerve were conducted. The skin temperature of the hand was maintained at 32 °C or above. All NCS studies were conducted at a standard room temperature (25 °C). For the ring finger test, the median and ulnar sensory NCSs were conducted on the ring finger by stimulating the wrist, 14 cm from the recording electrode. In addition, needle electromyography (EMG) of the abductor digiti minimi (ADM), abductor pollicis brevis (APB), flexor carpi radialis (FCR), triceps, biceps brachii, and cervical paraspinalis muscles was performed to exclude cervical radiculopathy or other peripheral neuropathies. The severity of CTS was classified according to Bland’s scale^11^ (Table 1).

**Table 1:** The severity of carpal tunnel syndrome.

Severity	Sensory NCS	Motor NCS	APB needle EMG
Mild: At least three of the following sensory and motor nerve conduction	14 cm wrist stimulation, peak latency > 3.7 ms 14 cm wrist stimulation, the peak latency: proximal 7 cm > distal 7 cm Transcarpal 5 cm short-segment latency: onset latency > 1.3 ms, peak latency > 1.5 ms 14 cm SNAP amplitude: 16–20 uV Conduction block greater than 50% in wrist palm stimulation if 14 cm stimulation amplitude ≥ 20 uV	Distal latency > 4.2 ms CMAP amplitude: 4.1–4.5 mV	Normal
Moderate: Mild PLUS at least two of the following	Wrist stimulation (14 cm) SNAP amplitude 6–15 uV Conduction block greater than 50% at wrist & palm stim. If SNAP ≥ 10 uV with 14 cm wrist stimulation	CMAP amplitude 2.1–4 mV	Fibrillation ( ±) Abnormal MUAP with intermediate interference pattern
Severe: Moderate PLUS	SNAP amplitude ≤ 5 uV	CMAP amplitude ≤ 2 mV	Fibrillation ( ±) Abnormal MUAP with discrete activity or single unit pattern

*NCS* nerve conduction study, *CMAP* compound motor action potential, *SNAP* sensory nerve action potential.

### Ultrasound examination

Ultrasound examination of the median nerve was performed in the outpatient clinic with the UGEO WS80A ultrasound system and a 5- to 13-Hz broadband linear transducer (Samsung Medison, Hongchun, Korea)^4^. Ultrasound settings were kept constant throughout the study. Ultrasound examinations were performed by a physiatrist with more than five years of experience with musculoskeletal ultrasound. The examiner was blinded to the results of NCS. Participants were imaged in the supine position with their elbow extended, forearm supinated, and shoulder in neutral position^4^.

**Figure 2:**
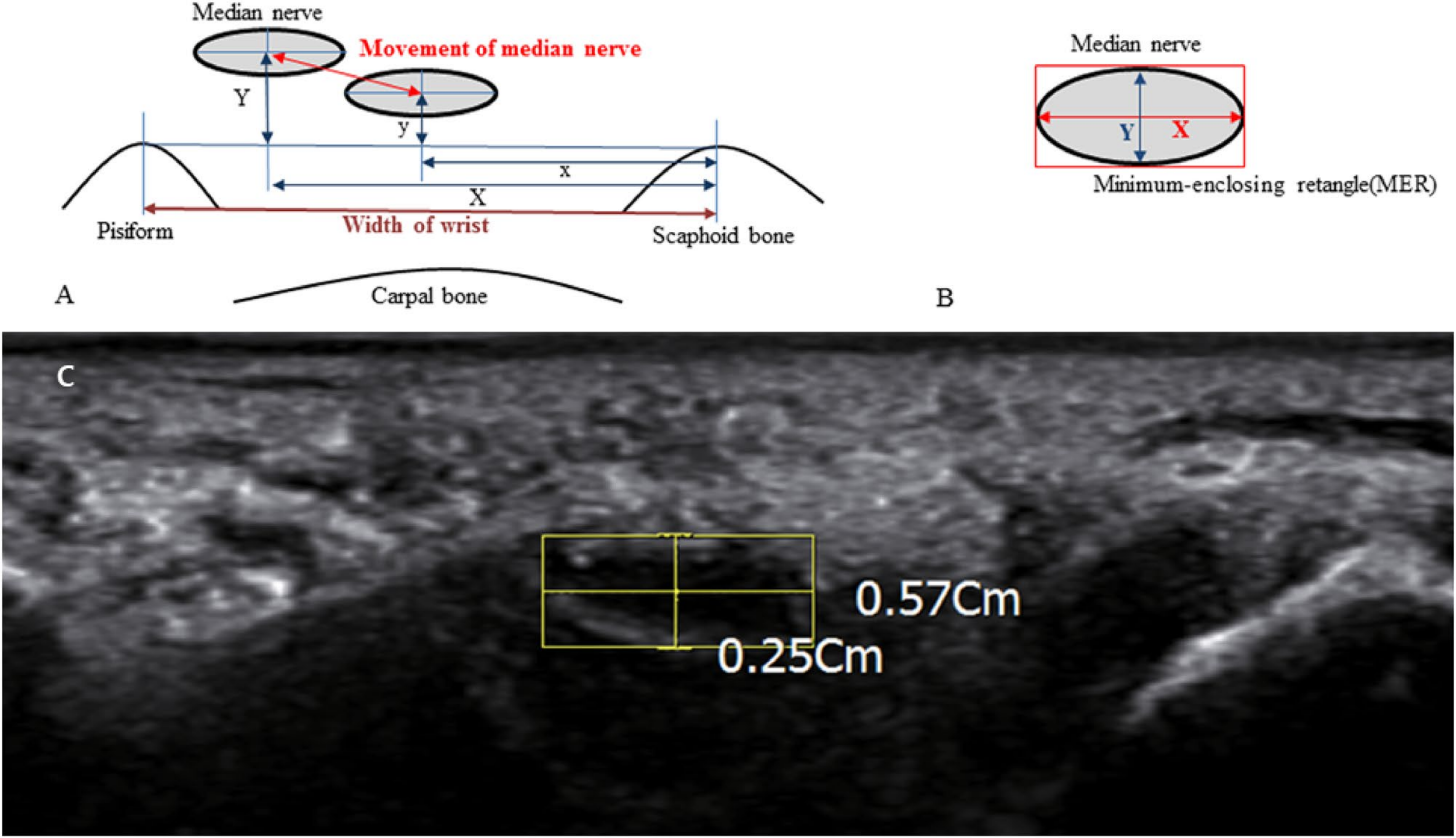
(**A**) Ultrasound measurement of transverse median nerve displacement in response to wrist and finger movement. To calculate the maximal change value of the median nerve displacement in response to finger and wrist movements, the maximal and minimal displacement of the median nerve from the scaphoid tubercle were chosen during six motions (the maximal displacement of the median nerve from the scaphoid tubercle = $\sqrt {{\text{X}}^{2} + {\text{Y}}^{2} } ;$ the minimal amplitude of the median nerve from the scaphoid tubercle = $\sqrt {{\text{x}}^{2} + {\text{y}}^{2} }$). The maximal change in the median nerve displacement was defined as: $\sqrt {\left( {X - x} \right)^{2} + \left( {{\text{Y}} - {\text{y}}} \right)^{2} }$. (**B**,**C**) The measurement method of the aspect ratio of the minimum-enclosing rectangle (MER). The aspect ratio of the MER was defined as $\frac{X \;(Major \;axis\; of\;MER)}{{Y\;\left( {Minor \;axis \;of\; MER} \right)}}$.

To study the transverse movement and deformation of the median nerve, cross-sectional images of the carpal tunnel were obtained by placing the transducer at the proximal carpal tunnel (Fig. 2A)^4,5^. The proximal carpal tunnel was defined as the area between the pisiform tubercle and the scaphoid^4,5^. To prevent anisotropy, the transducer was kept perpendicular to the median nerve, without additional pressure, to minimize compression of the tissue in the carpal tunnel.

Cross-sectional ultrasound images were obtained during the following maximal voluntary movements of the finger and wrist joints:

1. Thumb, second, and third finger flexion: The wrist was held in a neutral position while the relevant finger or thumb was moved to maximal flexion.
2. Grasp: The wrist was held in a neutral position, while all four fingers and the thumb were moved to maximal flexion, making a fist.
3. Wrist ulnar deviation with finger extension: The motion of the wrist from the neutral position to maximal ulnar deviation was examined, with the fingers maintained in full extension.
4. Wrist radial deviation with finger extension: The motion of the wrist from the neutral position to maximal radial deviation was examined, with fingers the maintained in full extension.

All patients were asked to perform maximal voluntary motion in each finger and wrist joint to minimize bias due to varying forces. After ultrasonography evaluation, all participants with CTS were treated with a single injection of a steroid (3 ml solution containing 0.5 ml of 20 mg triamcinolone and 2.5 ml of 0.5% lidocaine) around the median nerve at the wrist, delivered by the same physiatrist. Ultrasound examinations of participants CTS were performed before and two weeks after the injection.

### Image analysis

All recorded images were saved in a digital form and evaluated using Preview (INFINITT, Seoul, South Korea)^4,12,13^. First, we captured an ultrasound image of the median nerve in the resting state with the finger extended and wrist in the neutral position. During each voluntary movement, if the median nerve did not move for more than 5 s, an ultrasound image was captured again, given that it was the end of the median nerve motion. Using a digital measurement tool within Preview, the median nerve was outlined manually, using a continuous boundary trace along the echogenic boundary of the nerve, before the following measurements were made.

To analyze the deformation of the median nerve, the cross-sectional area (CSA) and aspect ratio of the minimum-enclosing rectangle (MER) of the median nerve were measured^5^. The CSA of the median nerve was measured by outlining the inner margin of the hyperechoic epineurium^14^. The aspect ratio of the MER was defined as the ratio of the major axis divided by the minor axis of this rectangle (Fig. 2B,C)^5^. When measuring the transverse carpal ligament (TCL), the ultrasound transducer was placed transversely at the carpal tunnel outlet of the wrist^15^.

The maximal change in the median nerve area was defined as the difference between the maximal and minimal areas of the median nerve from among six wrist and finger movement trials.

The maximal change in the median nerve aspect ratio of the MER was defined as the difference between the maximal and minimal aspect ratios of the MER of the median nerve from among six wrist and finger movement trials.

In addition, to analyze the motion of the median nerve during voluntary movements of the finger and wrist joint, the maximal change value of the median nerve displacement in response to the finger and wrist movements was calculated. To calculate the maximal change value, we first measured the distance between the center of the median nerve and the imaginary lines of the scaphoid bone landmark (radioulnar direction, x). Secondly, we measured the distance between the median nerve and the transverse line connecting the tubercles of pisiform and scaphoid (dorsopalmar direction, y) (Fig. 2A). The The displacement of the median nerve from the scaphoid tubercle at the end of each movement was calculated as $\sqrt {{\text{x}}^{2} + {\text{y}}^{2} }$^5^. Next, the maximal and minimal displacement of the median nerve from the scaphoid tubercle were chosen among the six motions. (The maximal median nerve displacement from the scaphoid tubercle = $\sqrt {{\text{X}}^{2} + {\text{Y}}^{2} }$; the minimal median nerve displacement from the scaphoid tubercle = $\sqrt {{\text{x}}^{2} + {\text{y}}^{2} }$). Finally, the maximal change in the median nerve displacement was defined as $\sqrt {\left( {X - x} \right)^{2} + \left( {{\text{Y}} - {\text{y}}} \right)^{2} }$ (Fig. 2A)^5^.

All measurements were obtained twice, and the average value of the two measurements was recorded.

### Normalization of measurements

The maximal change value of the median nerve displacement was normalized with respect to the width of the wrist, which was defined as the distance from the tubercle of scaphoid to the pisiform (Fig. 2B). Moreover, the maximal change values of the aspect ratio of the MER, and the median nerve area were normalized to the aspect ratio of the MER and the median nerve area in the neutrally positioned wrist with finger extension. The normalized results were presented as Normalized Units (NU) where 1 NU = 1% of the normalized length. The distance between the scaphoid and pisiform tubercle was measured using ultrasound images^5^. Thus, if the distance from the scaphoid to the pisiform tubercle was 4 cm and the actual movement of the median nerve was 0.4 cm, the measurement would be expressed as 10 NU, i.e., 10% of the wrist width. Moreover, if the area of the median nerve in neutral position was 20.0 mm^2^, and the absolute change value of the median nerve area was 4.0 mm^2^, the measurement would be expressed as 20 NU, i.e., 20% of the median nerve area in the wrist in a neutral position.

### Reliability of median nerve measurements

To assess intra- and inter-rater reliability, ultrasound data for 10 healthy participants and 10 participants with CTS (pre-injection) were analyzed. Intra- and inter-rater reliability of ultrasonographic measurement was determined using intra-class correlation coefficients (ICCs) with corresponding 95% confidence intervals (CIs). For the assessment of the intra-rater reliability, a single rater, blinded to the results of the electrophysiologic study, analyzed the aspect ratio of the MER and CSA of the median nerve in different ultrasound images two times at different time-points. For the assessment of the inter-rater reliability, two raters, blinded to the results of the electrophysiologic study, analyzed the aspect ratio of the MER and CSA of the median nerve in 10 healthy participants and 10 participants with CTS in different ultrasound images two times at different time-points,. We achieved satisfactory intra- (ICC = 0.986; 95% CI 0.973–0.992) and inter-rater reliability (ICC = 0.968; 95% CI 0.939–0.983).

### Statistical analysis

Statistical analyses were performed using SPSS version 22 (IBM Corp., Armonk, New York, USA). To compare the healthy participants and patients with CTS, statistical analysis was initially performed using an independent t-test, based on the results of the Kolmogorov–Smirnov test. The Pearson’s chi-square test was used to assess the sex ratio in the healthy and CTS groups. The pre- and post-injection states in participants with CTS were assessed using multivariate logistic regression (forward stepwise regression) to identify the parameters affecting the outcomes (Table 4). The results were presented as mean ± standard deviation. P-values < 0.05 were considered statistically significant.

## Results

### Participant characteristics

The mean age of the healthy participants was 59.85 ± 13.41 years and that of the patients with CTS was 56.46 ± 13.46 years (p = 0.123). The sex composition of both groups was similar (p = 0.168), as was the difference between the left and right sides (p = 0.506; Table 2).

**Table 2:** Baseline characteristic of healthy subjects and patients with carpal tunnel syndrome (CTS).

	Healthy subjects	Patients with CTS	*P* value
Age (year)	64.57 ± 11.52	58.50 ± 11.38	0.123
Gender (M:F)	8 : 6	7 : 17	0.168
Direction (R:L)	5 : 9	12 : 12	0.506

*M* male; *F* female; *R* right; *L* left.

### Pre-injection ultrasound findings

**Figure 3:**
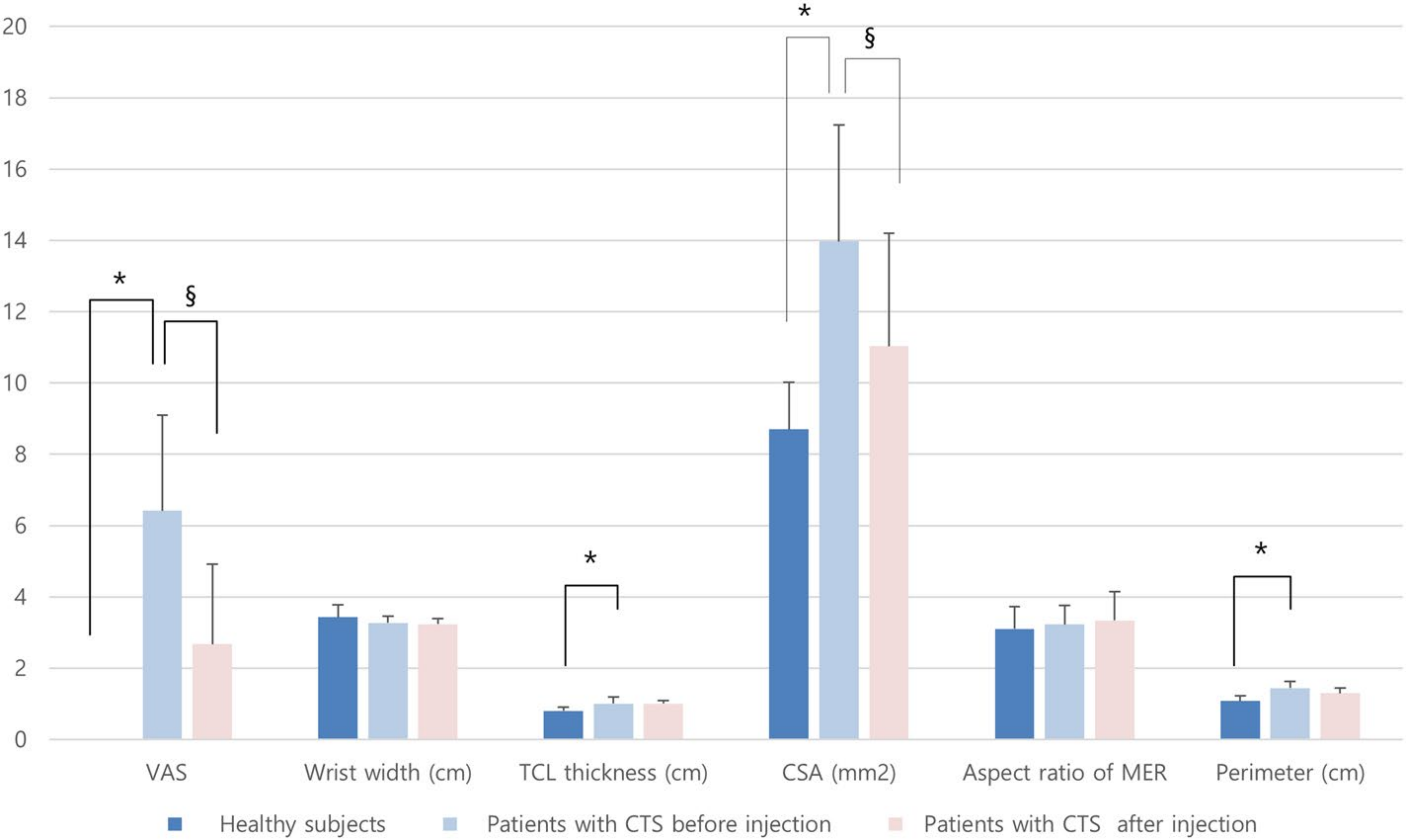
Ultrasound findings for healthy participants and patients with carpal tunnel syndrome before and after steroid injection. *CSA* the cross-sectional area at the proximal portion of the carpal tunnel; *TCL* trans-carpal ligament; *MER* minimum-enclosing rectangle. *P < 0.05 compared with ultrasound findings in healthy subjects. ^§^P < 0.05 compared with ultrasound findings in patients with CTS before injection.

The cross-sectional area of the median nerve at the proximal carpal tunnel was 8.70 ± 1.31 mm^2^ in healthy participants, and 13.97 ± 3.27 mm^2^ in patients with CTS before injection (Table 3; p = 0.0001). In addition, there was a significant difference in the TCL thickness at the carpal tunnel outlet of the wrist between the healthy participants and patients with CTS before the injection (p < 0.05). The normalized maximal change in the median nerve displacement, CSA, and perimeter values also showed significant differences between the healthy participants and patients with CTS before the injection (p < 0.05; Fig. 3; Table 3). In contrast, there was no difference between these groups with respect to the aspect ratio of the MER (p > 0.05).

**Table 3:** Clinical and ultrasound findings of healthy subjects and patients with carpal tunnel syndrome.

	Healthy subjects	Patients with CTS before injection	Patients with CTS after injection
VAS	0.00 ± 0.00	6.42 ± 2.67*	2.67 ± 2.24^§^
Wrist width (cm)	3.43 ± 0.34	3.27 ± 0.19	3.24 ± 0.15
TCL thickness (cm)	0.08 ± 0.01	0.10 ± 0.02*	0.10 ± 0.01
CSA (mm^2^)	8.70 ± 1.32	13.97 ± 3.27*	11.03 ± 3.17^§^
Aspect ratio of MER	3.10 ± 0.62	3.23 ± 0.53	3.34 ± 0.80
Perimeter (cm)	1.08 ± 0.14	1.44 ± 0.19*	1.30 ± 0.14
NU of displacement (%)	18.67 ± 5.59	13.02 ± 4.42*	14.39 ± 5.46
NU of area (%)	43.40 ± 3.28	24.64 ± 11.76*	32.05 ± 11.93^§^
NU of aspect ratio of MER (%)	64.57 ± 7.02	58.43 ± 21.54	52.86 ± 19.34
NU of perimeter (%)	36.55 ± 4.10	20.71 ± 12.35*	21.84 ± 7.28

*CTS* carpal tunnel syndrome; *TCL* transverse carpal ligament; *CSA* cross-sectional area of median nerve at proximal carpal tunnel; *MER* minimum-enclosing rectangle.

*P < 0.05 compared with ultrasound findings in healthy subjects.

^§^P < 0.05 compared with ultrasound findings in patients with CTS before injection.

### Post-injection ultrasound findings

There was a significant difference in the visual analog scale (VAS) scores recorded before and after the injection in patients with CTS (p < 0.001). Significant differences were also noted in the CSA and perimeter of the median nerve values recorded before and after the injection in patients with CTS (p < 0.001; Table 3).

There were no significant differences in the change in the normalized maximal distance, normalized maximal change in the MER aspect ratio, or maximal change in the perimeter of the median nerve due to finger and wrist movements among patients with CTS before and after the injection (p > 0.05). However, we noted a statistically significant difference in the normalized maximal change in the median nerve CSA values during hand and wrist movements recorded before and after injection in patients with CTS (p < 0.05; Table 3).

**Figure 4:**
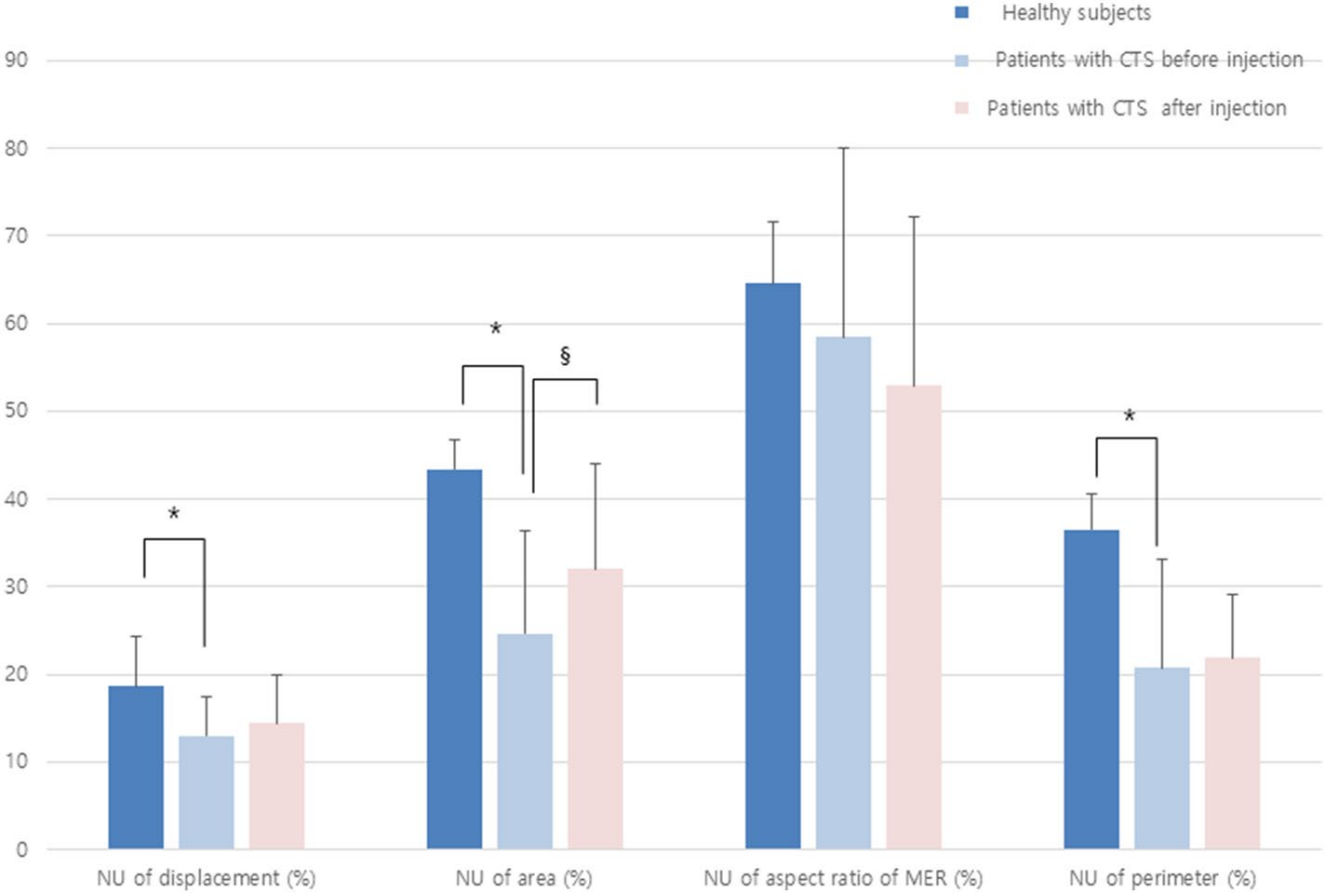
Diagram of the normalized parameters of the median nerve recorded during ultrasound examination. *CTS* carpal tunnel syndrome; *NU* normalized unit. *P < 0.05 compared with ultrasound findings in healthy subjects. ^§^P < 0.05 compared with ultrasound findings in patients with CTS before injection.

In multivariate logistic regression analysis, only the difference in the normalized maximal change in the median nerve CSA during finger and wrist movements correlated with the administration of the steroid injection (p < 0.05; Fig. 4; Table 4).

**Table 4:** Multivariate logistic regression analysis for assessing significantly changed parameter after steroid injection in patients with mild stage of CTS.

Parameter	β coefficient	Standard error	Odds ratio (95% CI)	*P* value
NU of area (%)	0.304	0.487	2.166 (0.001–0.024)	0.036

*CI* confidence interval; *NU of area* normalized unit of maximal change of value of median area.

## Discussion

The pathophysiology of CTS is multifactorial. Although the etiology of increased carpal tunnel pressure associated with CTS is unclear, experimental evidence suggests that compression or inflammation may be the causative factor^16^. Currently, ultrasonography is used for diagnosing CTS, with a focus on detecting focal swelling of the median nerve in the carpal tunnel^17^. However, previous studies have suggested that a decrease in the transverse plane movement of the median nerve in ultrasonography is a characteristic feature of patients with CTS^18,19^.

In the present study, median nerve deformation and movement significantly differed between patients with CTS and healthy participants. This finding is in contrast with the findings of previous studies. In a recent ultrasonography-based study, Park et al.^4^ suggested that only the nerve swelling-related parameter changed during early-stage CTS (Bland’s scale 1–2) without significant changes in the motion of the median nerve during finger and wrist movements. This may be because of the within-group differences among patients with CTS in the study by Park et al. In our study, patients with CTS and Bland’s scale score 1–3 were included. Therefore, the significant changes in the motion of the median nerve during finger and wrist movements between healthy participants and patients with CTS may be attributed to the enrollment of patients with a relatively more severe stage of CTS.

Our results indicate significant differences in median nerve deformation and motion during finger and wrist movements between the healthy participants and patients with CTS. However, only the normalized maximal change in the median nerve CSA during finger and wrist motions was significantly correlated with the administration of the steroid injection in patients with CTS. After the steroid injection, the normalized maximal change in the median nerve CSA increased. In patients with CTS, the median nerve is believed to be considerably swollen; therefore, the magnitude of change in the median nerve CSA may accordingly decrease during finger and wrist movements. Therefore, the decrease in the swelling of the median nerve s after steroid injection may lead to an increase in the normalized maximal change in the median nerve CSA during finger and wrist movements. Nevertheless, the administration of a steroid injection could not trigger significant changes in the normalized maximal change in the displacement of the median nerve during finger and wrist movements. These findings suggest that a decrease in the maximal change in the median nerve displacement, which may be related to connective tissue fibrosis (i.e., SSCT), is not associated with the administration of the steroid injection in patients with CTS.

Although previous studies have reported on the effect of steroids in the treatment of various fibrosis conditions^20,21^, improvement in CTS on administration of a steroid injection may be associated with the improvement in nerve swelling rather than in fibrosis of the SSCT.

Additionally, we examined CTS patients with Bland scale scores of 1–3 in this study, and the change in the morphology and motion of the median nerve during finger and wrist movements showed significant differences compared to those in the healthy participants. However, in previous studies, which included only CTS patients with Bland’s scale scores of 1–2, only the change in the median nerve morphology during finger and wrist movements was significantly different compared to that in the healthy participants. Considering the differences in these findings, we assumed that in patients with more advanced CTS, the motion of the median nerve during finger and wrist movements, which is indicative of the fibrosis of the SSCT indirectly, can change more frequently. Therefore, the swelling of the median nerve due to compression may be involved in early-stage CTS.

There are several limitations of this study. First, the sample size was small. Second, we did not investigate the change in the median nerve during wrist flexion and extension, although the highest intra-carpal tunnel pressure is generated during these movements and maximal hand grasping motion. However, a previous study has reported that maximal hand grasping movement alone was sufficient to alter the deformation of the median nerve in patients with CTS^4^. In addition, the transverse movement of the median nerve tends to be induced by radial and ulnar deviations of the wrist rather than flexion or extension of the wrist joint. However, this study did not aim to identify the degree of deformation change or the absolute value of the intra-carpal pressure; rather, the study aimed to identify the parameters affected by the administration of the steroid injection. For the investigation of this objective, we did not need to assess wrist flexion and extension in the present study. Third, we only identified the motion and deformation of the median nerve in patients with CTS. Further studies including histological analyses or animal experiments, such as those evaluating changes in the flexor tendons together those in the median nerve in patients with CTS, may provide evidence regarding the pathogenesis of early-stage CTS. Fourth, the present study included a single follow-up assessment performed two weeks after steroid injection. It is plausible that additional assessments may aid in revealing the transverse movement of the median nerve, indicative of SSCT fibrosis. Therefore, further research is required.

Last, the present study was not the first study to evaluate the CSA and mobility of the median nerve after steroid injection^22,23^. However, the two studies reported previously followed a different protocol to evaluate the same outcomes. To assess median nerve mobility, participants in the previous studies were instructed to repeatedly flex and extend their fingers and wrist, while the transducer was maintained over the distal wrist crease. Mobility was rated as “normal (2),” “slightly decreased (1),” or “decreased (0).” “Normal” mobility was defined as the median nerve diving deep into the flexor tendons during finger and wrist flexion. However, this method is problematic, as it may be less accurate compared to the methods used in the present study, which assessed the exact distance of interest. Hence, we believe that the parameters used in the present study are more suitable for examining the movement of the median nerve and associated SSCT fibrosis. In addition, owing to the crudeness of the mobility score (3-grade system) used in the previous studies, the reported mobility scores and CSA seemed to improve after steroid injection. However, a more granular assessment of the distance or normalized distance performed in the present study has revealed no statistically significant difference. Therefore, despite the limitations of our study, our findings regarding parameters related to median nerve compression and SSCT fibrosis provide meaningful preliminary evidence.

## Conclusion

In patients with mild CTS, the median nerve significantly decreased in size after the steroid injection compared to that before steroid injection However, statistically significant changes were noted only in the median nerve morphology during finger and wrist movements after steroid injection. Considering the difference in the ultrasound findings in this study compared to the findings of previous studies, it can be assumed that in patients with more advanced CTS, the change in the motion of the median nerve during finger and wrist movements, which is indicative of fibrosis of the SSCT indirectly, can occur more frequently. Therefore, median nerve swelling due to compression may be involved in early-stage CTS pathogenesis. However, further studies are required to overcome the limitations of this study and to elucidate the pathogenesis of CTS.
